# Supplementary material for: The Role of NMDA Receptor Partial Antagonist, Carbamathione, as a Therapeutic Agent for Transient Global Ischemia
Source: Biomedicines. 2023 Jul 3;11(7):1885. doi: 10.3390/biomedicines11071885 (PMC10377037; doi:10.3390/biomedicines11071885)
Supplement: Supplementary file 1 [file biomedicines-11-01885-s001.zip › biomedicines-2382612-supplementary.pdf]

Supplementary Table S1: List of reagents used for cell culture

**Reagents***	**Company and Catalogue Number**
F-12 K media	ATCC Cat# ATCC$^{(R)}$30-2004^{TM}
Trypsin-EDTA solution	ATCC Cat# ATCC$^{(R)}$30-2101^{TM}
Horse serum	ATCC Cat# ATCC$^{(R)}$30-2040^{TM}
Fetal bovine serum	ATCC Cat# ATCC$^{(R)}$30-2020^{TM}
Rat pheochromocytoma (PC) PC-12 cell line	ATCC Cat# ATCC$^{(R)}$ CRL-1721.1^{TM}
Poly-D-Lysine	Sigma Cat# P6407-5MG
Glutamate (L-Glutamic acid monosodium salt hydrate)	Sigma Cat# G5889-100G
Mammalian Protease inhibitor cocktail	Sigma Cat# P8430-5ML
ATP Bioluminescent Assay kit	Promega Cat# G7573
RIPA Lysis and Extraction Buffer	G-Biosciences Cat# 786-490
Halt^{TM} Phosphatase inhibitor	Thermo Scientific Cat# 1861277
Bio-red Protein Assay Dye Reagent Concentrate	Biored Cat# 5000006
96 Well plate	VWR Cat# 10861-666
6 Well plate	VWR Cat# 10062-892

*All the reagents purchased in year 2018-2019 (between 01/15/2018 to 12/15/2019).

Supplementary Table S2: List of antibodies used for western blotting

**Antibody***	**Company and Catalogue Number**
Anti-GRP78 antibody	Abcam (Waltham, MA 02453, USA), Cat# ab21685
Anti-Hsp 70antibody	Abcam (Waltham, MA 02453, USA), Cat# ab31010
Anti-Hsp 27 antibody	Abcam (Waltham, MA 02453, USA), Cat# ab12351
Anti-Caspase-12 antibody	Abcam (Waltham, MA 02453, USA), Cat# ab62484
Anti-IRE1 antibody	Abcam (Waltham, MA 02453, USA), Cat# ab37073
Anti-ATF4 antibody	Abcam (Waltham, MA 02453, USA), Cat# ab85049
Anti-XBP1 Antibody	Abcam (Waltham, MA 02453, USA), Cat# ab37152
Anti-GAPDH antibody	Cell Signaling Technologies (Danvers, MA 01923, USA.), Cat# 5174S
Anti-Akt antibody	Cell Signaling Technologies (Danvers, MA 01923, USA.), Cat# 4691S
Anti-Phospho Akt (P-Akt) antibody	Cell Signaling Technologies (Danvers, MA 01923, USA.), Cat# 4060S
Anti-Bax antibody	Cell Signaling Technologies (Danvers, MA 01923, USA.), Cat# 2772S
Anti-Beclin-1 antibody	Cell Signaling Technologies (Danvers, MA 01923, USA.), Cat# 3738S
Anti-eIF2α antibody	Cell Signaling Technologies (Danvers, MA 01923, USA.), Cat# 2103S
Anti-P-eIF2α antibody	Cell Signaling Technologies (Danvers, MA 01923, USA.), Cat# 9721S
Anti-P-STAT 3 antibody	Cell Signaling Technologies (Danvers, MA 01923, USA.), Cat# 9145S
Anti-Cleaved Caspase- 3 antibody	Cell Signaling Technologies (Danvers, MA 01923, USA.), Cat# 9664S
Anti-CHOP/GADD153 antibody	SantaCruz Biotechnology (Dallas, Texas 75220 U.S.A.), Cat# sc-793
Anti-Bcl-2 (N-19) antibody	SantaCruz Biotechnology (Dallas, Texas 75220 U.S.A.), Cat# sc-492
Anti-ATF6 antibody	Novus Biologicals:; Centennial, CO 80112, USA (another name:Imgenex), Cat# IMG-273/ NBP1-40256
Secondary goat anti-mouse antibody	LI-COR Bioscience (Lincoln, Nebraska USA 68504-0425), cat#925-32210
Secondary goat anti-rabbit antibody	LI-COR Bioscience (Lincoln, Nebraska USA 68504-0425), cat#925-32211

*All the antibodies purchased in year 2018-2019 (between 01/15/2018 to 12/15/2019).
